# The Legal Status of Hospital-Trained Nurses

**Published:** 1907-06-08

**Authors:** Herbert W. G. Macleod

**Affiliations:** 17 Upper Wimpole Street, W.


### THE LEGAL STATUS OF HOSPITAL-TRAINED NURSES.

Dear Sir,—I shall be obliged if you can inform me where I can obtain a book on the legal status of hospital-trained and certificated nurses and of their legal responsibilities to the public. I do not mean those under the Midwives Act. Thanking you in anticipation. Yours faithfully,

Herbert W. G. Macleod.

17 Upper Wimpole Street, W., May 31.

[The book entitled "How to Become a Nurse; the Nursing Profession, how and where to Train" (Scientific Press), contains a chapter on the principal laws affecting nurses.—Ed. The Hospital.]

